# Prevalence of psychiatric disorders, comorbidity patterns, and repeat offending among male juvenile detainees in South Korea: a cross-sectional study

**DOI:** 10.1186/s13034-017-0143-x

**Published:** 2017-01-18

**Authors:** Johanna Inhyang Kim, Bongseog Kim, Bung-Nyun Kim, Soon-Beom Hong, Dong Woo Lee, Ju-Young Chung, Ji Young Choi, Bum-Sung Choi, Young-Rim Oh, Miwon Youn

**Affiliations:** 1 Division of Child and Adolescent Psychiatry, Department of Psychiatry, Seoul National University College of Medicine, 101 Daehak-no, Seoul, Chongno-gu 03080 Republic of Korea; 2 Department of Psychiatry, Sanggye Paik Hospital, Inje University College of Medicine, 1342 Dongil-ro, Seoul, Nowon-gu 01757 Republic of Korea; 3 Department of Psychiatry, Pusan National University Yangsan Hospital, 20 Guemo-ro, Mulgeum-eup, Yangsan, Gyeongsangnam-do 50612 Republic of Korea; 4 Department of Social Welfare, Yongin Songdam College, 61 Dongbu-ro, Yongin, Cheoin-gu 17145 Republic of Korea; 5 Youn’s Therapy Counseling Center, Yulim Building 3F, 119 Bangbae-ro, Seoul, Seocho-gu 06682 Republic of Korea

**Keywords:** Juvenile detainees, Psychiatric disorder, Alcohol use disorder, Comorbidity, Repeat offending

## Abstract

**Background:**

High rates of psychiatric disorders and comorbidities have been reported in juvenile detainees, and both phenomena are thought to contribute to repeat offending. However, research on this topic has been limited in Asian countries, like South Korea. The purpose of this study was to examine the prevalence of psychiatric disorders, comorbidity patterns, and the relationship between psychiatric disorders and repeat offending among a cross-section of youths detained in a male juvenile detention center in South Korea.

**Methods:**

One hundred seventy-three juvenile detainees were recruited. The distribution of psychiatric disorders within the sample was estimated by applying criteria from the *Diagnostic and Statistical Manual of Mental Disorders IV*. Logistic regression was used to assess significant comorbidity patterns and relationships between psychiatric disorders and repeat offending.

**Results:**

In all, 90.8% of the detainees had at least one psychiatric diagnosis, and 75.1% had psychiatric comorbidities. The most common psychiatric disorder was alcohol use disorder, followed by conduct disorder and attention-deficit hyperactivity disorder. Among the comorbidities present, alcohol use disorder with disruptive behavior disorder was the most common combination. The presence of two psychiatric disorders was associated with a higher rate of recidivism, and alcohol use disorder was also associated with repeat offending when combined with disruptive behavior disorders, but not with anxiety disorders, major depression, or psychotic disorders.

**Conclusions:**

Juvenile detainees evidence high rates of psychiatric disorders and comorbidities. Assessment of and intervention in psychiatric disorders, especially alcohol use disorder and comorbid alcohol use disorder with disruptive behavior disorders, may help prevent further offenses.

## Background

Many studies have reported high rates of psychiatric disorders in juvenile detainees. Previous studies have reported that 40 to 90% of juvenile detainees have at least one psychiatric disorder [1–6], which accounts for about a three- to four-fold increase in the prevalence of psychiatric illnesses compared to the general population [7–9]. Some psychiatric disorders in youths, like conduct disorder (CD) and substance use disorder (SUD), are thought to be related to more severe antisocial behavior, more violent offending, and increased criminal behavior in adulthood [10, 11]. Screening and recognition of mental problems in juvenile offenders may help identify risk factors for continued criminal behavior, facilitate treatment, and eventually lead to more positive outcomes [12]. However, the proportion of detainees who receive proper screening or intervention for mental health problems is small in South Korea. To promote awareness of this issue, the magnitude of the psychiatric problems experienced by juvenile offenders must be investigated via epidemiological research.

Although extensive research on the prevalence of psychiatric disorders in juvenile offenders has been conducted in Western countries, epidemiological research concerning this issue is limited in South Korea. A Chinese study reported that 80.2% of male detainees met criteria for any psychiatric disorder, and 38.8% were diagnosed with at least two disorders [13]. A study of juvenile offenders in Malaysian prisons demonstrated that almost all offenders had at least one diagnosable psychiatric disorder [14]. A previous study targeting 1155 juvenile detainees in South Korea reported high rates of depression, paranoia, antisocial personality, and hypomania using the Minnesota Multiphasic Personality Inventory–Adolescent (MMPI-A) scale [15]. However, no study has yet estimated the prevalence of psychiatric disorders in juvenile detainees in South Korea using criteria from the *Diagnostic and Statistical Manual of Mental Disorders* (DSM) or International Classification of Diseases (ICD) [15].

Comorbidity is common among juvenile offenders [1, 3, 16–18]. The reported comorbidity rate ranges from 20 to 63%, and several studies have shown that SUD plus disruptive behavioral disorders (DBDs) is the most common comorbidity combination [3, 17]. However, the detailed profile of comorbidity patterns among juvenile detainees is unclear, as some studies have focused on only a few selected disorders, like depression or SUDs [16, 19–21]. Others combined psychiatric disorders into broader categories, like internalizing disorders, SUDs, or DBDs [3, 20]. Furthermore, the patterns of comorbidity among juvenile offenders have not been studied in Asian countries like South Korea [11].

The assessment of psychiatric disorders and comorbidity patterns among juvenile offenders is important, as both are thought to be linked to repeat offending. Various studies have studied the association between psychiatric disorders and repeat offending [21–25], but the specific disorders that predicted repeat offending differed among studies, and positive findings were reported with regard to SUDs [23], affective disorders [23], oppositional defiant disorder (ODD) [24], and CD [21, 25]. Some of these previous studies did not take into account comorbidity [24, 25], and this may have affected the results, considering the high rate of comorbid disorders. McReynolds and colleagues reported that SUDs and DBDs, along with their comorbidity, predicted repeat offending [23]. Anxiety disorder predicted repeat offending only when it was comorbid with DBDs, and affective disorders were associated with repeat offending only when combined with SUDs in males [23]. However, this study used broad diagnostic grouping categories and did not investigate individual psychiatric disorders. Other studies have also reported that psychiatric comorbidity predicted criminal repeat offending, but there was no information regarding which psychiatric comorbidity combination contributed to these results [22, 26].

We conducted this cross-sectional study to answer three research questions. The first purpose of this study was to investigate the prevalence of psychiatric disorders, and the second was to determine the comorbidity patterns in juvenile detainees in South Korea. We further examined the relationship between psychiatric disorders and repeat offending, as well as the association between specific psychiatric comorbidity patterns and repeat offending.

## Methods

### Participants

Detainees were recruited from a male juvenile detention center in Seoul, South Korea, during the period of December 2015 to January 2016. According to Article 32 Section 3 of the Juvenile Act, juvenile offenders in South Korea are sentenced to one of 10 dispositions after trial in juvenile court. The 8–10th dispositions involve detainment for various durations. We excluded detainees sentenced to the 8th disposition which orders detainment for less than 1 month, and the 200 detainees sentenced to a 6-month (9th disposition) or a 2-year (10th disposition) detainment were included. Detainees over 19 years of age were excluded (n = 27), which left a total of 173 participants for this study, ranging in age from 15 to 19 years (Table 1). Participants were eligible regardless of psychiatric diagnosis, state of drug or alcohol intoxication, or fitness to stand trial. Exclusion criteria included refusal or inability to cooperate, or inability to understand the study procedures.

**Table 1:** Demographic and judicial characteristics of detainees

Characteristic	Detainees (n = 173)
Age (years), mean (SD)	17.5 (1.1)
School drop- out, N (%)	42 (24.3)
Yearly family income > $25,000, N (%)	104 (60.1)
Paternal education ≥ college education, N (%)	25 (14.5)
Maternal education ≥ college education, N (%)	20 (11.6)
Living arrangements, N (%)	
With both parents	57 (32.9)
With a single parent	97 (56.1)
No parents	19 (11.0)
Recidivism, N (%)	154 (89)
Type of index offense, N (%)	
Property crime	86 (49.7)
Violent crime	68 (39.3)
Sex crime	34 (19.7)
Drug crime	1 (0.6)
Domestic violence	1 (0.6)
Traffic offenses	42 (24.3)
Obstruction of justice	7 (4.0)
Drunk driving	2 (1.2)
Others	20 (11.6)

*SD* standard deviation

Written informed consent was obtained from the participants and guardians (in case of participants under the age of 18) after they were provided with a sufficient explanation of the study. This study protocol was approved by Sanggye Paik Hospital’s institutional review board (IRB No. SGPAIK 2015-06-022-002).

### Procedures

The psychiatric diagnoses were confirmed using the Mini International Neuropsychiatric Interview (MINI), which is a short, structured psychiatric interview that can detect a wide range of DSM-IV and ICD-10 psychiatric disorders [27]. The MINI consists of 19 modules that explore 17 Axis I of the DSM-IV disorders, as well as the risks of suicide and antisocial personality disorder. It has been validated against structured interviews including the Structured Clinical Interview for DSM-III-R and the World Health Organization-designed Composite International Diagnostic Interview [27, 28]. The MINI has shown fair inter-rater reliability, in that all kappa values were >0.75; it also has demonstrated good test–retest reliability, in that 61% of the kappa values were >0.75 [27]. It has been applied to the assessment of psychiatric disorders in various criminal justice settings [29, 30]. The Korean version has well-established validity and reliability [31]. The interview was conducted by clinical psychologists with a master’s degree after 4 h of training on the administration of MINI.

Psychiatric disorders were grouped into broader categories for analyses: DBDs (CD, ODD, ADHD), SUDs (alcohol use disorder and other SUDs), and any anxiety disorder (panic disorder, social phobia, obsessive–compulsive disorder, post-traumatic stress disorder, and generalized anxiety disorder). Psychotic disorders and major depression did not belong to any category and were included in analyses individually.

Demographic data (age, school drop-out, annual family income, parental education, living arrangements) and judicial data (type of crime, recidivism) was collected using self-report questionnaires. Repeat offending was defined as conviction of any type of criminal offense more than once. The type of index offense was defined according to the criminal law and special laws of South Korea. Property crimes include theft, fraud and embezzlement. Violent crimes include robbery, physical assaults, and blackmailing.

### Statistical analyses

Descriptive statistics were used to summarize participants’ demographic and judicial characteristics, and to estimate the prevalence of each psychiatric disorder.

A series of logistic regression analyses was conducted between diagnostic categories to identify comorbidity patterns. We adjusted for covariates that were found to be significantly associated with having comorbidities (p < 0.1) in univariate regression models. Potential covariates included age (continuous variable), socioeconomic status (SES; annual income of more than $25,000 or less than $25,000), maternal and paternal education level (having a college education or more or having less than a college education), school drop-out status (yes or no), living situation (living with no parent or with at least one parent), and violent crime commission (yes or no). Covariates were added to hierarchical multivariable logistic regression models.

The relationship between number of psychiatric disorders and repeat offending was analyzed using logistic regression. The association between each psychiatric disorder and repeat offending was also analyzed by applying logistic regression. Univariate regression was used to investigate the association between repeat offending and the potential covariates that have been previously mentioned. Covariates that showed a significant association (p < 0.1) were further added to the hierarchical multivariate logistic regression models (covariates in block 1, psychiatric disorder in block 2).

None of the multivariate linear regression models revealed multicollinearity (defined as variance inflation factor, VIF > 5) among the independent variables, and goodness-of-fit was evaluated using the Hosmer–Lemeshow test.

We further investigated the relationships between specific comorbidity patterns and repeat offending rates using logistic regression analyses. As there were many patterns of comorbidity, we selected the psychiatric disorder (s) that was (were) found to be significantly associated with repeat offending in the previous analyses, and analyzed the association of the various comorbidity patterns of this disorder (s) with repeat offending. Repeat offending was the dependent variable, and subgroups defined by dividing the detainees according to comorbidity pattern (e.g. alcohol use disorder + DHD, alcohol use disorder without ADHD, ADHD without alcohol use disorder, others) were entered as independent variables. The models were further adjusted for covariates that were found to be associated with repeat offending in the previous analyses.

All statistical analyses were performed using SPSS ver. 22.0 software (SPSS Inc., Chicago, IL, USA), and a two-tailed *p* value < 0.01 (0.05/5 diagnostic categories) was considered significant.

## Results

In total, 157 (90.8%) participants had at least one psychiatric diagnosis. Alcohol use disorder was the most common diagnosis, followed by conduct disorder. Among the 104 (60.1%) with SUDs, 100 (57.8%) had alcohol use disorder and 8 (4.6%) had other SUDs. Among the 123 (71.1%) with DBDs, 95 (55.5%) had CD, 61 (35.3%) had ADHD, and 14 (8.1%) had ODDs. Thirty detainees (17.3%) had major depression, 2 (1.2%) had dysthymia, 35 (20.2%) and 47 (27.2%) had an episode of hypomania or mania, respectively. A total of 44 (25.4%) had anxiety disorders, and among them 5 (2.9%) fulfilled the diagnostic criteria for post-traumatic stress disorder. The number of participants with a psychotic disorder was 19 (11.0%), 47 (27.2%) had tic disorders. The pattern of comorbidities is presented in Table 2. Among potential covariates, only annual family income was associated with having psychiatric comorbidities (p < 0.1), and this was added to the model as a covariate. Alcohol use disorder with DBDs was the most common combination, accounting for 46.2% of the detainees, followed by DBDs with anxiety disorders (22.5%). DBDs were significantly associated with alcohol use disorder and anxiety disorders. Alcohol use disorders showed significant association with DBDs. Psychotic disorders were associated with anxiety disorders. Anxiety disorders had an increased risk to be associated with DBDs, psychotic disorders and major depression. Major depression was associated with psychotic disorders and anxiety disorders.

**Table 2:** Comorbidity patterns across psychiatric diagnoses

Comorbid disorder	DBD	AUD	Psychotic disorder	Anxiety disorder	Major depression
DBD, N (%)					
N (%)		80 (46.2)	16 (9.2)	39 (22.5)	25 (14.5)
AOR (95% CI)		2.83 (1.44–5.59)^**^	2.32 (0.64–8.45)	4.33 (1.57–11.99)^**^	3.07 (1.01–9.33)^*^
AUD					
N (%)			14 (8.1)	26 (15.0)	22 (12.7)
AOR (95% CI)			2.46 (0.83–7.31)	1.17 (0.57–2.39)	2.41 (1.00–5.82)
Psychotic disorder					
N (%)				11 (6.4)	8 (0.5)
AOR (95% CI)				4.24 (1.53–11.71)^**^	4.04 (1.43–11.37)^**^
Anxiety disorder					
N (%)					20 (11.6)
AOR (95% CI)					10.00 (4.03–24.81)^***^
Major depression					
N (%)					
AOR (95% CI)					

*DBD* disruptive behavior disorder, *AUD* alcohol use disorder, *AOR* adjusted odds ratio

* p < 0.05

** p < 0.01

*** p < 0.001

The univariate regression analyses of the associations between demographic/judicial characteristics and repeat offending revealed that only school drop-out was significantly associated (p < 0.1) with repeat offending. Table 3 summarizes the odds ratios (ORs) for repeat offending according to each individual psychiatric disorder. Alcohol use disorder showed a nominally significant association with repeat offending (p = 0.018). The number of comorbidities among detainees ranged from 2 to 11 and 130 (75.1%) had comorbidities. Table 3 shows the relation between number of comorbidities and repeat offending rate. Having 2 psychiatric disorders increased the repeat offending rate (p = 0.009), but having one psychiatric disorder or three or more psychiatric disorders was related torepeat offending.

**Table 3:** Adjusted odds ratios for repeat offending according to psychiatric disorder

Diagnosis	Repeat offending			
	OR (95% CI)	p value	AOR^a^ (95% CI)	p value
1 psychiatric disorder	1.47 (0.33–6.52)	0.615	1.56 (0.34–7.05)	0.566
2 psychiatric disorders	10.67 (1.81–15.28)	0.009	13.50 (1.32–19.14)	0.008
3 or more psychiatric disorders	1.17 (0.45–3.04)	0.749	1.04 (0.39–2.73)	0.942
DBDs	2.48 (0.94–6.54)	0.066	2.63 (0.98–7.05)	0.055
CD	1.18 (0.70–4.81)	0.218	0.188 (0.71–4.99)	0.206
ODD	0.72 (0.15–3.49)	0.681	0.74 (0.15–3.66)	0.711
ADHD	1.20 (0.43–3.35)	0.722	1.20 (0.43–3.38)	0.727
AUD	3.39 (1.22–9.42)	0.019	3.43 (1.22–9.62)	0.019
Psychotic disorder	1.06 (0.22–4.97)	0.946	0.88 (0.18–4.30)	0.877
Any anxiety disorder	0.95 (0.32–2.81)	0.925	0.87 (0.29–2.62)	0.804
PTSD	2.08 (0.22–19.67)	0.522	2.71 (0.26–28.43)	0.406
Major depression	0.76 (0.23–2.48)	0.651	0.69 (0.21–2.31)	0.551
Mania	1.27 (0.45–3.57)	0.647	1.65 (0.57–4.78)	0.356
Hypomania	1.06 (0.33–3.41)	0.925	1.00 (0.30–3.25)	0.993

*OR* odds ratio, *CI* confidence interval, *AOR* adjusted odds ratio, *DBD* disruptive behavior disorder, *CD* conduct disorder, *ODD* oppositional defiant disorder, *ADHD* attention-deficit/hyperactivity disorder, *AUD* alcohol use disorder, *PTSD* post-traumatic stress disorder

^a^Adjusted for school drop-out

The association between repeat offending and alcohol use disorder with various comorbid patterns is presented in Table 4. Only alcohol use disorders plus DBDs showed a significant association with repeat offending (odds ratio 5.29, 95% confidence interval 1.69–16.54, p = 0.004).

**Table 4:** Adjusted odds ratios for repeat offending in alcohol use disorder according to comorbidity

Comorbidity combination	Recidivism			
	OR (95% CI)	p value	AOR^a^ (95% CI)	p value
AUD and CD				0.025
AUD+ CD (n = 66)	3.70 (1.17–11.72)	0.026	3.81 (1.18–12.32)	0.025
AUD only (n = 34)	10.00 (1.21–82.61)	0.033	10.17 (1.21–85.15)	0.032
CD only (n = 30)	2.73 (0.68–10.92)	0.156	2.83 (0.69–11.56)	0.148
Without AUD and/or CD	1 (Reference)		1 (Reference)	
AUD and ADHD				
AUD+ ADHD (n = 38)	2.92 (0.74–11.54)	0.125	2.95 (0.74–11.76)	0.126
AUD (n = 62)	4.92 (1.27–18.99)	0.021	4.88 (1.25–19.11)	0.023
ADHD (n = 23)	1.67 (0.41–6.74)	0.474	1.61 (0.39–6.69)	0.509
With AUD and/or ADHD	1 (Reference)		1 (Reference)	
AUD and DBD				
AUD+ DBD (n = 80)	5.29 (1.69–16.54)	0.004	5.64 (1.75–18.15)	0.004
AUD only (n = 20)	8.143 (0.942–70.409)	0.057	8.11 (0.91–71.93)	0.060
DBD only (n = 43)	4.18 (1.15–15.21)	0.030	4.46 (1.19–16.72)	0.027
Without AUD and/or DBD	1 (Reference)		1 (Reference)	
AUD and psychotic disorder				
AUD+ Psychotic disorder (n = 14)	2.79 (0.33–23.38)	0.345	2.23 (0.26–19.47)	0.468
AUD only (n = 86)	3.47 (1.16–10.40)	0.026	3.64 (1.20–11.03)	0.023
Psychotic disorder only (n = 5)	0.86 (0.09–8.37)	0.894	0.89 (0.09–9.10)	0.922
Without AUD and/or psychotic disorder	1 (Reference)		1 (Reference)	
AUD and anxiety disorder				
AUD+ anxiety (n = 26)	1.92 (0.49–7.56)	0.353	1.80 (0.45–7.26)	0.409
AUD only (n = 74)	5.92 (1.56–22.39)	0.009	5.84 (1.53–22.32)	0.010
Anxiety only (n = 18)	2.00 (0.40–10.02)	0.399	1.73 (0.34–8.96)	0.512
Without AUD and/or anxiety disorder	1 (Reference)		1 (Reference)	
AUD and major depression				
AUD+ major depression (n = 64)	2.18 (0.45, 10.61)	0.334	2.14 (0.43, 10.60)	0.351
AUD only (n = 36)	3.93 (1.20–12.84)	0.024	3.73 (1.13, 12.33)	0.031
Major depression only (n = 28)	0.66 (0.12, 3.65)	0.629	0.38 (0.06, 2.52)	0.315
Without AUD and/or major depression	1 (Reference)		1 (Reference)	

*OR* odds ratio, *CI* confidence interval, *AOR* adjusted odds ratio, *AUD* alcohol use disorder, *CD* conduct disorder, *ADHD* attention-deficit/hyperactivity disorder, *DBD* disruptive behavior disorder

^a^Adjusted for school drop-out

## Discussion

This is the first study to investigate the prevalence of psychiatric disorders, comorbidity patterns, and their relationships with repeat offending in juvenile detainees in South Korea. There was a high rate of psychiatric disorders and comorbidities among the juvenile detainee population, as is the case with Western countries [2, 3]. The percentage of detainees with at least one psychiatric disorder was 90.8%, and although direct comparisons are problematic due to differences in samples and measurement methods, this figure was high compared to the reported rate of 15–38% among the general adolescent population [32–34]. Similarly, the rates of alcohol use disorders and CD were much higher than those witnessed in the general population, as a national cohort in the US reported lifetime rates of 11.8 and 13.2% for alcohol abuse and dependence in adolescence, respectively, and a meta-analysis of 47 studies reported a 2.1% prevalence rate for CD [35, 36]. In addition, as was the case in previous studies, these two were the most common disorders [2, 6]. Compared with a meta-analysis of 3401 male adolescents sampled from studies from 10 different countries (United States, Canada, Japan, Russia, the Netherlands, Belgium, the United Kingdom, Denmark, Austria and Finland), our study reported a higher prevalence of ADHD (35.3 vs 13.5%) and psychotic disorders (11 vs 1.4%), and a lower prevalence of SUDs other than alcohol use disorder (4.6 vs 45.8%) [5]. This may be due to differences in the study population in terms of diagnostic tools (self-reported questionnaires vs. interviews), diagnostic criteria (DSM-III-R vs. DSM-IV), sample size, race, and age range. The low rate of SUDs other than alcohol use disorder matches the findings of [37], who reported the lifetime prevalence of illicit drug use among the general Korean adolescent population to be 0.4%, which was much lower than the observed rate among adolescents in other countries [38, 39].

Comorbidity seems to be the rule, rather than the exception, in justice settings [40, 41]. Psychiatric professionals in the judicial system should be aware of the significant comorbidity patterns, and look for one when another is present (e.g. look for anxiety disorders when a DBD is present). The combination of alcohol use disorders and DBDs was the most common comorbidity combination observed in previous studies [3, 14] as well as in this one. The comorbidity of SUDs and CD has been well-studied, as some genetic studies suggest a heritable risk of substance abuse in families with antisocial personality disorder and adoption studies have also reported a greater risk of SUDs in individuals with CD [42]. As comorbid CD and SUD is related to more severe antisocial behavior and more violent offending [10, 11], clinicians should be aware of this potentially dangerous combination.

Alcohol use disorder was not significantly comorbid with major depression. This result is inconsistent with previous studies that reported significant associations between major depression and SUDs, including alcohol use disorder [3]. The non-significant association may be partially explained by the exclusion of female detainees in this study, as affective disorders and SUD may be more strongly linked in females than in males [43]. The stronger association between affective disorders and SUD in females compared with males may be due to the decreased reliability of reported depressive symptoms in males [43]. Nevertheless, as comorbid depression and SUDs may lead to more substance dependence, an increased number of substances used regularly, and an increase in the incidence of suicide planning, the detection and treatment of both conditions is important for improving treatment outcomes [44, 45].

Repeat offending was associated with the presence of psychiatric comorbidities. Among the individual psychiatric disorders, only alcohol use disorder showed a nominally significant association with repeat offending. When looking at the comorbidity patterns with alcohol use disorders, there was a significant association when alcohol use disorders were combined with DBDs. However, there was no significant association when alcohol use disorders were combined with ADHD, anxiety disorders, major depression, and psychotic disorders. McReynolds et al. reported a significant association between repeat offending and SUDs plus DBDs, which matches the results of this study, but they also reported that SUDs plus affective disorders increase repeated offending, which disagrees with the present results. However, direct comparisons between study results are difficult, as McReynolds study used the category of affective disorders, but we only investigated the combination with major depression, as the rate of hypomania and mania was very high in our data set [23]. Indeed, the high prevalence of hypomanic and manic episodes in our sample may have been caused by confusion between these phenomena and ADHD.

Contrary to previous studies, not any psychiatric disorder belonging to the DBD category increased repeat offending [46]. There have been controversial results regarding the relationship between ODD or ADHD and repeat offending, but many results have reported a positive relationship between CD and repeat offending [46, 47]. The discrepancy with our results may be due to differences in sample size, the definition of repeat offending, or the types of crime included. Other factors could be under-reporting by juvenile detainees or under-detection of repeat offenses by the police. Cohn et al. reported similar results, in that they found no relationship between persistent offending and ODD/CD [48]. As the development of conduct problems is influenced by temperament and environmental factors, the frequency of conduct problems can vary according to temporary changes in the environment [49]. However, DBDs were significantly related to repeat offending when comorbid with alcohol use disorders. This finding suggests that the assessment of comorbidity patterns, not only single psychiatric disorders, is important for the prediction of repeat offending. As repeat offending was assessed in a retrospective manner, a causal relationship cannot be determined and further prospective studies with larger sample sizes are warranted.

The practice parameters for youths in juvenile detention and correctional facilities developed by the American Academy of Child and Adolescent Psychiatry (AACAP) recommended that all youth receive screening at entrance and continued monitoring for mental problems [49]. In South Korea, the resources for providing services for the identification of and intervention in the psychiatric problems experienced by juvenile offenders are limited. Regarding treatment, currently there is only one medical protection facility for juvenile offenders in South Korea that can provide psychiatric treatment. Furthermore, this facility accommodates only 60 patients at once and there is no full-time board-certified psychiatrist present. As juvenile offenders often come from deprived backgrounds, with little access to and use of healthcare in the community, opportunities for intervention in the juvenile justice system have the potential to make a significant impact on public health terms [49, 50]. As this study shows, there is a high rate of psychiatric disorders among those in the juvenile justice system of South Korea, and development of assessment protocols and intervention programs is necessary.

This study has some noteworthy limitations. The relatively small sample size may have underpowered our results. Furthermore, this study was conducted using a cross-sectional design; thus, the causality between psychiatric disorders and repeat offending remains undetermined. We only included male subjects, as the targeted juvenile detention center housed males only, and this may limit the generalizability of the results to both genders within the juvenile justice system. Likewise, because we conducted the study inside the detention center, we were unable to obtain information from informants other than the detainees themselves. This may have led to the underreporting of some psychiatric symptoms, especially externalizing behaviors. We used the MINI to diagnose psychiatric disorders, but this does not fully cover child and adolescent psychiatric diagnoses. We had no information on the time spent in detention, so we were unable to consider the effects of this on psychiatric diagnoses. Finally, we only included detainees from a single detention center, and further large-scale studies using a prospective design that includes detainees from various areas and detention centers are warranted.

## Conclusions

Almost all the juvenile detainees in this particular detention center in South Korea had at least one psychiatric disorder and a substantial proportion of detainees had at least one comorbid psychiatric disorder. The prevalence of SUD was 57.8%, that of major depression was 17.3%, and that of DBDs was 71.7%. These findings highlight the need to diagnose and intervene in psychiatric disorders and comorbidities in the juvenile detention system, especially when they concern alcohol use disorder plus DBDs. For further research, we suggest prospective studies with large sample sizes to determine the impact of psychiatric disorders and comorbidities on the long-term outcomes of detainees, especially in adulthood.

## Abbreviations

MMPI-A: Minnesota Multiphasic Personality Inventory–Adolescent
DSM: Diagnostic and Statistical Manual of Mental Disorders
ICD: International Classification of Diseases
SUD: substance use disorder
ODD: oppositional defiant disorder
CD: conduct disorder
MINI: Mini International Neuropsychiatric Interview
SES: socioeconomic status
ADHD: attention-deficit/hyperactivity disorder
